# 1246. Clinical isolates of *Pseudomonas aeruginosa* Harbor Preexisting Changes in TonB-dependent Receptors Associated with Decreased Susceptibility to Cefiderocol

**DOI:** 10.1093/ofid/ofab466.1438

**Published:** 2021-12-04

**Authors:** Stephanie L Egge, Shelby Simar, An Q Dinh, Blake Hanson, Truc T Tran, Cesar A Arias, William R Miller, William R Miller

**Affiliations:** 1 University of Texas Health Sciences Center Houston, Houston, Texas; 2 University of Texas Health Science Center, Houston, TX; 3 Center for Antimicrobial Resistance and Microbial Genomics, UTHealth, Houston, TX, Houston, Texas; 4 CARMiG, UTHealth and Center for Infectious Diseases, UTHealth School of Public Health, HOU, TX ; Molecular Genetics and Antimicrobial Resistance Unit and International Center for Microbial Genomics, Universidad El Bosque, BOG, COL, Houston, Texas; 5 Center for Antimicrobial Resistance and Microbial Genomics, UTHealth, Houston, TX

## Abstract

**Background:**

Cefiderocol (FDC) is a novel siderophore cephalosporin that retains activity against MDR gram-negative bacteria. In *P. aeruginosa* (*PA*), FDC utilizes TonB-dependent receptors (TBDR) PirA, PiuA, or PiuD to enter the periplasmic space. We recently reported a clinical *PA* isolate that developed elevated MICs to FDC associated with mutations in genes encoding TBDRs in the absence of prior exposure to FDC. In this study, we investigated the frequency of TBDR mutations not associated with cefiderocol exposure among clinical stains of *P*A recovered from 1999 to 2018 in a large hospital system in Houston, TX.

**Methods:**

A total of 212 clinical isolates of *PA* were screened for mutations in TBDR pathways (*pirA*/*pirRS* and *piuA*/*D*) via whole genome sequencing. Strains with gene mutations predicted to significantly alter protein function (insertion, deletion, or frameshift) were selected for phenotypic characterization. *PA* PA01 and 4 clinical *PA* strains lacking changes in the TBDR genes served as controls. FDC susceptibility testing was performed on Mueller-Hinton agar by Kirby-Bauer disc diffusion (DD). Diameters were measured at 18 h and 48 h to assess for the emergence of colonies inside the zone of inhibition. Breakthrough colonies were isolated on cetrimide agar, and DD studies were performed to determine FDC susceptibility.

**Results:**

6.1% of isolates (13/212) had preexisting mutations in the TBDR genes, including indels in *piuA* (n=2) and *pirR* (n=2), and a frameshift mutation resulting in premature stop codon in *pirR* (n=9). DD showed that isolates with predicted changes in TBDRs had a significantly smaller diameter of inhibition, as compared to controls (Fig 1). Of the PiuA or PirR mutants, 3 of 13 demonstrated breakthrough colonies (Fig 2); while none of the control specimens showed breakthrough colonies. Subcultures of isolated breakthrough colonies yielded more homogenous populations of *PA* with relatively lower DDs than the original strain (Fig 2).

Figure 1

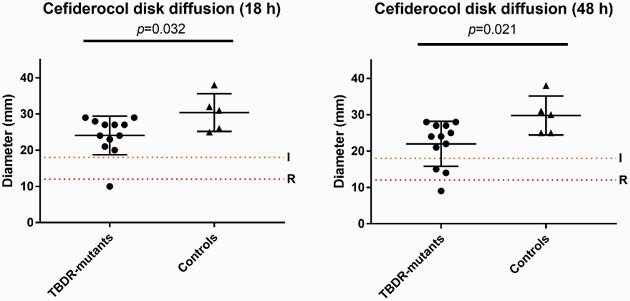

Cefiderocol disk diffusion diameters of P. aeruginosa isolates at (a) 18 hours growth and (b) 48 hours growth. P-values for student’s two-tailed t-test are given. Dotted lines represent cutoffs for intermediate and resistant per Clinical Laboratories and Standards Institute (CLSI) guidelines. ns, non-significant; TBDR, TonB dependent receptor.

Figure 2

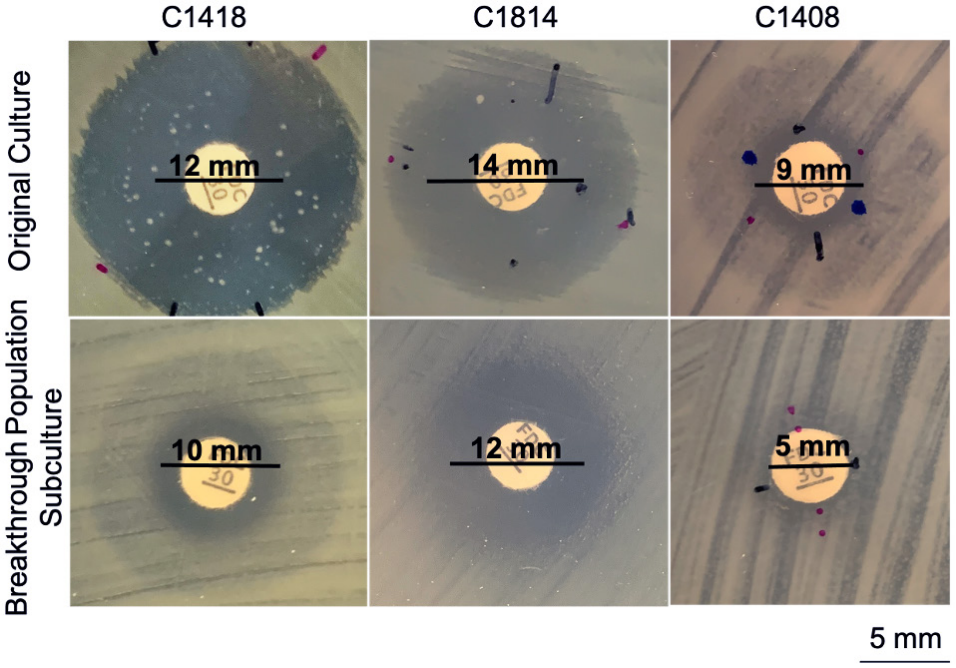

Disk Diameter resistance phenotypes suggestive of heteroresistance among P. aeruginosa strains containing TBDR mutations (row 1) and their subsequent breakthrough colony subculture resistance phenotypes (row 2) at 48 hour time points.

**Conclusion:**

Mutations in genes encoding TBDR are present in clinical isolates of *PA* that predate the approval of FDC and are associated with the emergence of reduced susceptibility to FDC.

**Disclosures:**

**Truc T. Tran, PharmD**, **Merck** (Grant/Research Support) **Cesar A. Arias, M.D., MSc, Ph.D., FIDSA**, **Entasis Therapeutics** (Grant/Research Support)**MeMed Diagnostics** (Grant/Research Support)**Merk** (Grant/Research Support) **William R. Miller, MD** , **Entasis Therapeutics** (Scientific Research Study Investigator)**Merck** (Grant/Research Support) **William R. Miller, MD** , Entasis (Individual(s) Involved: Self): Scientific Research Study Investigator; Merck (Individual(s) Involved: Self): Grant/Research Support

